# Association between public health emergencies and sexual and reproductive health, gender-based violence, and early marriage among adolescent girls: a rapid review

**DOI:** 10.1186/s12889-023-15054-7

**Published:** 2023-01-17

**Authors:** Shruti Shukla, Jessy Amarachi Ezebuihe, Janina Isabel Steinert

**Affiliations:** 1 TUM School of Social Sciences and Technology, Technical University of Munich, Munich, Germany; 2 The World Bank, Washington, DC USA; 3 Department of Social Policy and Intervention, University of Oxford, Oxford, UK

**Keywords:** Gender inequality, Public health emergencies, Adolescent girls, Sexual and reproductive health, Child marriage, Violence against women, COVID-19

## Abstract

**Background:**

As COVID-19 continues to impact lives and livelihoods around the world, women and girls are disproportionately affected. Crisis situations and related response measures, such as lockdowns, school closures, and travel restrictions, often exacerbate the adversities and human rights violations faced by adolescent girls. We conducted a rapid review to synthesise evidence on the impact of public health emergencies (PHEs) related to gender-based inequalities among adolescent girls.

**Methods:**

We systematically searched five major databases. Records were imported into the online screening tool *Rayyan,* and 10% of the records were triple screened for eligibility. We included qualitative, mixed-methods, and quantitative studies that assessed the relationship between PHEs and any of the following outcomes: (1) gender-based violence, (2) early/forced marriage, and (3) sexual and reproductive health. Due to the heterogeneity of included study designs, no meta-analysis was performed, and studies were summarised narratively.

**Findings:**

Out the initial 6004 articles, 11 studies met our eligibility criteria. Five of these assessed the impact of natural disasters and six were focused on consequences of the COVID-19 pandemic. Seven studies focused on the impact of PHEs on gender-based violence, three focused on sexual and reproductive health, and only one study looked at early marriage. The main impacts highlighted by the studies included (1) increases in physical, psychological, and sexual abuse, (2) increase in the occurrence of teenage pregnancy, (3) poor menstruation hygiene management, and (4) occurrence of early marriages. Mechanisms underlying these impacts were PHE-specific response strategies like home confinement, closure of schools, the worsening of families’ financial situation such as the inability to pay for school fees or day-to-day living costs, and the disempowerment of and increased workloads for adolescent girls.

**Conclusion:**

Although evidence on the impact of COVID-19 on gender-based violence, sexual and reproductive health, and especially forced or early marriage of adolescent girls is limited, results from studies on other PHEs indicate that during crises, these detrimental outcomes are exacerbated. Findings from our review have important implications for policies and programs providing life skills training, financial literacy training, credit support, and safe spaces for adolescent girls.

## Introduction

As of October 2022, the COVID-19 pandemic has caused more than 6.5 million deaths worldwide [1]. This estimate worsens when accounting for excess mortality associated with COVID-19 by combining the direct and indirect impacts of the pandemic. While men suffered higher COVID-19 mortality rates, women and girls bore the brunt of the social and economic consequences of the pandemic [2]. A recent study published in *The Lancet* aimed to quantify important gender disparities induced by the pandemic, revealing that 26% of women reported employment losses during the pandemic, compared to 21% of men [3]. Women’s income generation opportunities were disproportionately affected because of their high employment in the informal sector, which left them with little to no social security compensations during lockdown periods [3, 4]. In addition, the pandemic increased the load of unpaid care work for women and girls. Specifically, estimates suggest that women were more than twice as likely than men to forego income to care for others [3]. Lastly, mobility restrictions during the pandemic, linked financial and emotional distress, and the high acceptance levels of intimate partner violence (IPV) in some countries fuelled a “shadow pandemic” of violence against women and girls [2, 5]. Similar harmful gendered impacts were observed during previous pandemics and epidemics. For example, during the 2013–16 Ebola outbreak, women endured higher levels of unemployment than men, suffered greater negative health impacts, and were at increased risk of infection due to their designated gender role as caregivers [6, 7].

Building on this prior evidence, our review aims to examine the impacts of public health emergencies (PHEs) through a gender lens. A PHE is hereby defined as an occurrence whose scale, time, or unpredictability may pose a threat to public health and wellbeing, and disrupt necessary routine actions [8, 9]. PHEs consist of disease outbreaks, epidemics, pandemics, and natural disasters (e.g., earthquakes, floods). The frequency of natural disasters and extreme weather events has drastically increased since the 1970s and is expected to further increase due to global warming and sea level rise [10]. The detrimental impacts of climate change on human health have been described as “the biggest global health threat of the twenty-first century” [11], and the World Health Organisation estimates that climate change will cause 250,000 additional annual deaths between 2030 and 2050 [12]. PHEs exacerbate the risks and vulnerabilities for women and adolescent girls, including their deprivation of education, exposure to gender-based violence, early and forced marriage, and teenage pregnancies [13–16]. A thorough understanding of the impacts of PHEs on gender inequality is urgently needed considering that PHEs will likely become increasingly prevalent in the coming years.

Adolescent girls are particularly vulnerable to experiencing multiple and long-lasting harmful consequences of PHEs. Adolescence is a crucial stage of life and adversities experienced during adolescence can lead to poor mental health, sexual and reproductive health (SRH) problems, and chronic diseases throughout adulthood [17–21]. Adverse exposure to abuse in adolescence can also lead to intergenerational transmission of adversity to children. For example, empirical evidence suggests that children whose mothers faced violence when growing up are at increased risk for developing clinically significant emotional and behavioural problems [19]. Additionally, research suggests that underage girls forced to marry are more likely to have children with worse nutritional outcomes as well as fewer years of education and worse educational achievements [21]. Moreover, adolescent girls are at increased risk because they may face violence victimisation by both caregivers and intimate partners simultaneously. Adolescent girls may also assume greater domestic responsibilities than adolescent boys in the absence or death of a caregiver [22, 23]. Lastly, PHEs may also lead to girls’ school dropout, which is associated with a range of negative outcomes, including increased risk of child or early marriages, increased exposure to violence perpetrators at home, and increased poverty [24–26].

While policy advocates have repeatedly warned that the COVID-19 pandemic is likely to affect adolescent girls in all aspects of their daily lives by harming their safety, wellbeing, and health, [27], a systematic and comprehensive evidence base on the harmful gender impacts of PHEs specific to adolescent girls is to date missing. In the face of the current COVID-19 pandemic and its associated mitigation strategies like lockdowns and school closures, youth-specific knowledge on the impact of PHEs is urgently needed. Likewise, it is important to gain a nuanced understanding on how girls may be effectively protected against adversity during PHEs. Building on this, our rapid review aims to identify and synthesise evidence on the effects of PHEs on three key gender outcomes of the Sustainable Development Goal 5: (1) sexual and reproductive health, (2) gender-based violence (GBV), and (3) forced or early marriage among adolescent girls. Additionally, the review aims to build knowledge on the mechanisms underlying this relationship.

## Methods

### Search strategy

A rapid review is a streamlined variation of a systematic review that can be conducted within a shorter time frame. It aims to provide a succinct summary of available research to inform context-specific decision making and guideline recommendations for an urgent policy issue [28]. For this rapid review, we used the SPIDER (Sample, Phenomenon of Interest, Design, Evaluation, Research type) methodology to define key elements of the search strategy and eligibility criteria. The SPIDER tool offers an alternative to the more frequently applied PICO (Population, Intervention, Comparison, Outcome) tool as it adapts the PICO components to make them better suited for searching qualitative and mixed-methods studies and is suitable for non-interventional studies by targeting the ‘phenomenon of interest’ instead of the ‘intervention or exposure’ [29]. We further followed the Cochrane Rapid Review Interim guidelines for reporting the results [30, 31]. A review protocol specifying the search strategy and eligibility criteria was published via the Open Science Foundation on 17 November 2021.

We searched for published studies reporting the impact of PHEs on adolescent girls between the ages 10–19 years. We systematically searched Web of Science core, Scielo, BIOSIS, Pubmed, and Medline between 3 November 2021 and 22 December 2021, and updated the search on 2 June 2022.

Search terms were categorised based on the SPIDER methodology. These included (1) PHEs or phenomenon of interest (POI), covering search terms for pandemics, epidemics, and natural disasters; (2) gender-related outcomes including forced or early marriage, sexual and reproductive health, and gender-based violence; and (3) population group specific to adolescent girls. In addition, we used a snowball technique, which involves examine reference lists from published articles, to find additional studies to include.

### Inclusion and exclusion criteria

We included studies that reported any of the three specified outcomes listed above for adolescent girls aged 10 to 19 years in the context of a PHE. We included quantitative, mixed-methods, and qualitative studies but excluded review articles, gray literature, and case studies on the topic, given time and resource constraints. Furthermore, there were no restrictions in terms of the geographical setting of the studies or the publication date.

We excluded studies on the Zika virus disease as it has direct consequences for maternal and child health indicators and is thus endogenous to pregnancy. We further excluded diseases like HIV, TB, and malaria as they are endemic in certain countries and regions and do not fall under our definition of PHEs in terms of the suddenness and unpredictability of events. Another reason for excluding HIV/AIDS was that our outcomes of interest might be bidirectionally intertwined with HIV, for example, GBV victimisation increases the risk of contracting HIV. We also excluded conflict-related humanitarian situations like wars and acts of terrorism as they may involve different mechanisms of impact on the gender-related outcomes and are purely human-controlled events [32]. Furthermore, we excluded studies with only male participants and studies with girls and women in the age groups below 10 years and above 19 years, unless studies disaggregated results by age. Lastly, we excluded studies with no full text available during the full-text screening stage.

### Study screening and data extraction

As per the COCHRANE rapid review guideline [30], we used a standardised title and abstract screening form and full-text screening form to conduct a pilot exercise on a sample of ten abstracts and five full-text articles to calibrate and test the review forms. The screening was implemented using *Rayyan*, a free web tool designed to help researchers working on knowledge synthesis projects for deleting the duplicates and conducting title and abstract screening [33]. All reviewers (SS, JE, JIS) screened 10% of the titles and abstracts (same subset) with blinding. Any conflicts were resolved in a post-screening discussion. The remaining abstracts were screened by two reviewers (SS, JE) without double screening. For the full-text screening, two reviewers (SS, JE) screened the selected articles and then cross-checked each other's excluded articles for conflict resolution.

A sample data extraction form was designed and piloted with five full-text articles. During data extraction, JE extracted data using the piloted form and SS checked for correctness and completeness of extracted data. The data extraction form included the following information: title, author and year, country/setting of the study, study design and type of collected data, age of target population, sampling and recruitment procedures, total number of participants, exposure (i.e. type of PHE, duration of exposure), gender-related outcomes measured, detailed information on measures, key findings on links between PHEs and outcomes, underlying mechanism of link (if analysed/discussed).

### Data synthesis

The standard guidelines [30, 31] to tabulate and narratively synthesise the results were applied. We categorised the outcomes based on the impact of PHEs on three main gender inequality indicators: (i) gender-based violence, (ii) forced or early marriage, and (iii) sexual and reproductive health, but excluded child health indicators as these do not directly apply to the defined target population of adolescent girls. Furthermore, we summarised insights into the chain of reactions emerging during PHEs that may lead to gender inequitable outcomes.

### Risk of bias assessment

One reviewer (SS) assessed the study quality and risk of bias of the selected papers using validated tools specific to each study type. The quality of cross-sectional quantitative studies was assessed with the Joanna Briggs Institute (JBI) tool [34], and the Critical Appraisal Skills Programme (CASP) appraisal tool was used for qualitative studies [35]. For mixed-methods studies, both tools were applied. A second reviewer (JIS) verified these ratings.

## Results

### Included studies

**Fig. 1:**
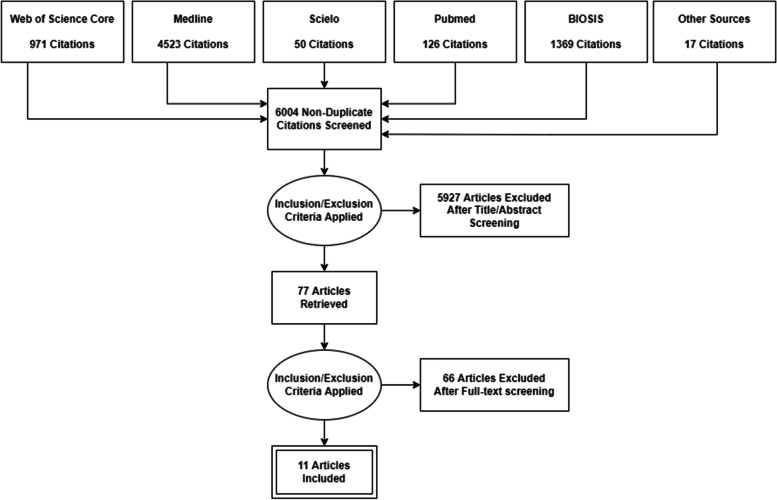
Flowchart of study selection

We screened 6004 unique records after deduplication. Of these, we excluded 5927 records after title and abstract screening. Sixty-six articles were excluded after full text screening because of one or more of the following reasons: (1) the age group of the examined population did not meet our eligibility criteria, (2) the study did not disaggregate the results by age and/or gender, (3) the outcome and/or PHE did not meet our eligibility criteria, (4) the type of publication did not meet our eligibility criteria, and (5) the study did not conduct a primary or secondary data analysis. Eleven studies met our eligibility criteria and are included in the synthesis below (see Fig. 1).

### Characteristics of included studies

The characteristics of the eleven included studies are presented in Table 1. Included studies were published between 2000 to 2022. The studies were implemented in Bangladesh (1), the United States (1), Haiti (2), Norway (1), Kenya (2), Indonesia (1), Brazil (1), in 19 different Sub-Saharan Africa countries (1), and in Sweden, the United States, Serbia, Morocco, and Vietnam (1). The PHEs studied were majorly COVID-19 (6); however, there were also earthquakes (2), floods (1), droughts (1), and hurricanes (1). The most common outcome studied was gender-based violence (7), followed by sexual and reproductive health (3). Only one study focused on child marriage. Although most studies were cross-sectional and quantitative (8), some were qualitative (2), and one was a mixed-methods study. The sample sizes varied largely, ranging from nine to 5,316 adolescent girls. The review summarises evidence from a total sample size of 13,836 adolescent girls.

**Table 1:** Characteristics of included studies

Study ID	Country	Study design	Study quality	Duration	Population age (years)	Sample size (females)	PHE	Outcome	Mechanisms underlying the effect of PHE on outcomes
Rashid and Michaud [36]	Bangladesh	Qualitative design using face-to-face in-depth interviews and informal discussions	Some concerns	1998	15–19	9	Flood	Sexual and reproductive health (privacy to bathe, use latrine, and maintain good menstrual hygiene)	Limited access to health services Disruption of social network Sharing a private space, like toilets and bathrooms, with men
Zulaika et al. [37]	Kenya	Panel data self-administered survey	High	May 2018 – March 2021	13–17	910	COVID-19	Sexual and reproductive health (pregnancy and sexual debut)	Closure of schools Disruption in schooling due to lockdown Reduction in household income
Temple et al. [38]	The United States	Cross-sectional face-to-face risk behaviour survey	Low	March 2009	14–18	584 447 (final sample)	Hurricane	Physical and sexual violence by a boyfriend	Intensity of exposure to traumatic stressor Substance abuse
Sloand et al. [39]	Haiti	Cross-sectional audio computer-based interviews	Some concerns	2011 –2013	12–17	78	Earthquake	Dating violence by boyfriend or ex-boyfriend and domestic violence by a family member	Low levels of education Young age Limited access to mental health services Social norms around intimate partner violence (IPV)
Cerna-Turoff et al. [40]	Haiti	Cross-sectional face-to-face household survey	High	2012	13–17	1,457	Earthquake	Physical, emotional, and sexual violence by a family or non-family member	Not reported
Epstein et al. [41]	19 Sub-Saharan African countries	Repeated face-to-face cross-sectional survey	High	2011 –2018	15–19	5,316	Drought	Physical, emotional, and sexual violence by intimate partner	Young age Lower social standing and inexperience with relationships Financial strain and food insecurity Stress and poor mental health conditions Disempowerment, unemployment, and economic dependence on partner
Augusti, Saetren, and Hafstad [42]	Norway	Cross-sectional web-based survey	Some concerns	June 2020	13–16	1,776	COVID-19	Physical, psychological, and sexual violence by a family or non-family member	Prior gender differences Family conflict Home confinement Previous experience with abuse (re-victimisation)
Karp et al. [43]	Kenya	Mixed-method study of phone-based survey and in-depth interviews	High	June –August 2020	15–19	407	COVID-19	Relationship quality, sexual behaviours, and contraception use	COVID-19 induced social restrictions Poverty, loss of financial resources or income Prior gender divide Economic instability Deteriorating relationship between partners
Kerekes et al. [44]	Sweden, the USA, Serbia, Morocco, Vietnam	Cross-sectional web-based survey	Some concerns	September 2020 –February 2021	15–19	3,120	COVID-19	Physical violence, threats, and sexual harassment by anyone	Socioeconomic status COVID-19 related restrictions Increased stress caused by physical distancing
Rahiem [45]	Indonesia	Qualitative design using face-to-face semi-structured in-depth interviews	High	March –December 2020	14–17	10	COVID-19	Child marriage	Marriage as an escape route or remedy for boredom Local customs Economic instability Lack of understanding of the impact and long-term consequences of underage marriage Peer pressure
Oliveira et al. [46]	Brazil	Cross-sectional face-to-face retrospective study	Some concerns	2016 –2020	14–18	169	COVID-19	Physical and sexual violence by a family or non-family member	Social isolation and breakdown of social networks Home confinement Closure of schools Patriarchal gender norms Young age Social norms around domestic violence

### COVID-19 and gender-based violence

Augusti, Saetren, and Hafstad [42] highlighted the associations between child abuse and known risk factors, both during and before the COVID-19 pandemic in Norway. Their study collected COVID-19-specific data in a subsample of adolescents (sample size: 1776 girls) that was included in a larger longitudinal study. The most common type of abuse during the COVID-19 outbreak was psychological abuse (10.3%), followed by witnessing of domestic violence (5.9%), and lowest for physical (3.2%) and sexual abuse (1.6%). Furthermore, the authors found that 11% to 24% of respondents (male and female combined) encountered psychological, physical, and sexual abuse for the first time in their lives during the pandemic and 47% of respondents were first exposed to online sexual violence during the pandemic. With regards to the association between pre- and post-pandemic abuse experience, the authors found prior victimisation to be the strongest risk factor associated with COVID-19-related violence. While the total rate of violence experienced by girls during the lockdown was lower (19.2%) than the previous year (37.9%), the authors explain that this was not surprising as the timeframe for reporting abuse experiences during COVID-19 was set to the past two months rather than the past twelve months.

Karp et al. [43] studied youth relationships during the COVID-19 pandemic using a phone survey in Kenya. The authors found that 17% of adolescent girls (15–19 years, sample size: 407) reported deterioration in the quality of their relationships during the pandemic. The social distancing regulations implemented to curb the spread of COVID-19 substantially reduced the time that non-cohabiting partners spent with each other. The reduced time with partners was the strongest predictor of changing relationship quality. Participants who described worse relationship quality were also more likely to experience IPV from their partner in the past month. Specifically, 21% of the adolescents and young women who reported worsening relationship quality also experienced IPV in the past 12 months.

Kerekes et al. [44] examined how the COVID-19 pandemic changed relationships, emotional wellbeing, and violence victimisation of adolescents (15–19 years, sample size: 3120 girls) in Sweden, the United States, Serbia, Morocco, and Vietnam, drawing on self-reported electronic survey data. Adolescent girls were more likely than boys to report physical assault (OR = 1.83, 95% CI = 1.55–2.16), defamation (OR = 1.31, 95% CI = 1.09–1.56), and being groped or touched in a sexual manner without their consent (OR = 1.77, 95% CI = 1.48–2.11) in the past 12 months. To quantify how the COVID-19 restrictions had affected abuse experiences, participants were asked to compare these experiences to those from prior to the outbreak of the pandemic. Adolescent girls reported an increased frequency for physical (OR = 1.49, 95% CI = 1.02–2.17) and sexual (OR = 1.49, 95% CI = 1.08–2.04) assaults since the pandemic affected their countries.

Oliveira et al. [46] studied the epidemiological profiles of violence against children (14–18 years, sample size: 169 girls) before and during the COVID-19 pandemic in Brazil. Data for this study was obtained from individual clinical records of child violence victimisation cases who received paediatric emergency assistance. Similar to Augusti, Saetren, and Hafstad [42], this study noted that the majority of the abuse had occurred at the victim’s home. Although emergency demands made by victims were reduced by March 2020, which the authors claim may be a result of the fear of COVID-19 infections, the rate of assistance increased from 0.10–0.36% in 2016–2019 to 0.673% in 2020.

### COVID-19 and child marriage

Rahiem [45] examined the cause of increased child marriage since the COVID-19 outbreak in Indonesia (14–17 years, sample size: 10 girls). Based on qualitative data analyses, some adolescent girls reported that they had personally decided to marry rather than having been forced because marriage would enable them to escape the dual pressure of school and home responsibilities. Other notable reasons for early marriage were local customs and beliefs that promote an early start of a family, lack of parents’ support and care during the pandemic, family economic problems, loneliness due to school closures, and peer pressure like seeing their friends getting married.

### COVID-19 and sexual and reproductive health

Karp et al. [43] also examined the influence of COVID-19 on the SRH of adolescent girls (15–19 years, sample size: 407 girls) in Kenya. The results of the in-depth interviews highlight that COVID-19-related restrictions reduced sexual contacts between partners due to a fear of viral transmission. In addition, prolonged school closures resulted in a loss of hope or interest in school for some respondents, thereby accelerating cohabitation with boyfriends, which was linked to an increased risk of early pregnancy. Furthermore, economic hardship fuelled by the pandemic increased girls’ economic dependency on partners or parents and their risk of engaging in transactional sex, which also increased the risk of unintended pregnancy.

Zulaika et al. [37] measured the effects of the pandemic on adolescent pregnancy and sexual behaviour. The authors compared the SRH of girls who completed their secondary education pre-pandemic in 2019 to girls who experienced disruption in their education due to the pandemic and graduated in 2021 (13–17 years, sample size: 910 girls). Their result showed that girls whose education was negatively affected by the pandemic had a two-fold increased risk of becoming pregnant prior to secondary school completion (adjusted risk ratio (aRR) = 2.11; 95% CI = 1.13–3.95), compared to girls who had graduated before the pandemic. The girls affected by education disruptions were also more likely to be sexually active (aRR = 1.28; 95% CI = 1.09–1.51) and less likely to report that their first sex was desired (aRR = 0.49; 95% CI = 0.37–0.65), relative to the girls who had graduated prior to the pandemic.

### Natural disasters and gender-based violence

Temple et al. [38] studied whether adolescents who were directly exposed to the Hurricane Ike in Texas, US displayed higher rates of physical and sexual teen dating violence, compared to adolescents who had been evacuated and were thus not directly exposed (14–18 years, sample size: 447 girls). The authors did not find any significant differences in dating violence between girls who had been evacuated and those who were exposed to the hurricane.

Sloand et al. [39] described the physical, psychological, and sexual violence experiences of internally displaced adolescent girls before and after the 2010 Haiti earthquake (12–17 years, sample size: 78 girls). The study was cross-sectional and pre-earthquake experiences were elicited through retrospective questions. Relying on audio- and computer-based interview methodology, the authors found that 50% of girls reported having experienced all three forms of violence before the earthquake and 64% reported violence experiences in the aftermath of the earthquake. About 22% of girls reported sexual abuse in similar percentages pre- and post-earthquake. The prevalence of physical and emotional abuse did not increase significantly after the earthquake.

Epstein et al. [41] studied the relationship between drought and IPV among adolescents (15–19 years, sample size: 5316 girls) and women in 19 Sub-Saharan African countries. Combining data from the Demographic and Health Survey with data from the Climate Hazards Group on InfraRed Precipitation with Station, they showed that adolescent girls who experienced droughts were significantly more at risk of having a controlling partner (marginal risk difference (RD) = 4.4, 95% CI 0.9–7.9) and experiencing emotional violence (marginal RD = 3.2, 95% CI 0.1–6.3), relative to those who did not experience any droughts. Similarly, drought exposure increased exposure to physical violence (marginal RD = 2.0, 95% CI 0.1–3.8). The authors contend that the inexperience of adolescents with relationships may exacerbate their vulnerability to IPV during periods of income instability and food insecurity.

Cerna-Turoff et al. [40] examined the impact of the internal displacement due to the 2010 Haitian earthquake on long-term physical, emotional, and sexual violence against adolescent girls (13–17 years, sample size: 1457 girls). The authors used a matching method to pair displaced and non-displaced girls who had similar characteristics before the earthquake. Girls who were internally displaced due to the earthquake did not have significantly higher odds of experiencing long-term physical, emotional, or sexual violence two years after the earthquake.

### Natural disasters and sexual and reproductive health

Rashid and Michaud [36] explored the implications of socio-cultural norms related to honour, shame, purity, and pollution for girls' experiences during the 1998 floods in Bangladesh (15–19 years, sample size: 9 girls). Using in-depth interviews and informal discussions, the authors found that one consequence of the floods was greater difficulty for most girls to secure their privacy when bathing or accessing latrines. Maintaining their privacy was also linked to protecting their reputation and self-respect as any indecent incident or harassment would bring their family shame and may ruin their marriage possibilities. Another finding was that the girls who had started menstruation were not able to maintain good menstrual hygiene in the aftermath of the flood. Due to strong social taboos around menstruation and the lack of clean water, it was difficult for girls to frequently wash and change their menstrual clothes.

### Mechanisms underlying the relationship between PHEs and gender-based inequalities

Our review identified a number of mechanisms through which PHEs may contribute to greater gender inequalities for adolescent girls [see Table 1]. First, PHE-specific response strategies like home confinement, social restrictions, and closure of schools may exacerbate existing vulnerabilities of adolescent girls. These strategies can lead to confinement of girls with their perpetrators or block pathways to report them [37, 39, 41]. They can also disrupt social networks leading to social isolation and increased psychological distress, such as depression and anxiety [41, 44, 46]. Furthermore, school closure may increase the risk of early marriage or pregnancy thus widening the gender gaps in education and future employment. For example, girls whose education was disrupted due COVID-19 pandemic were more likely to fall pregnant and be sexually active compared to girls who graduated pre-pandemic [37].

Second, PHEs may disrupt access to healthcare services [36, 39, 41]. Natural disasters may lead to physical obstruction in the availability of SRH products, such as feminine hygiene products, and pandemics may lead to disruption in the supply chain for SRH products, such as modern contraceptives. For example, two studies included in our analysis found that the SRH needs of girls faced increased jeopardy during PHEs due to limited access to health services and economic losses [36, 37]. Inaccessibility to SRH products like menstrual pads, contraceptives, and abortion services can lead to a range of negative health impacts ranging from infections to unwanted pregnancies or even mortality.

Third, PHEs often lead to substantial economic losses both at a national and a household level. Increased economic instability, food insecurity, and unemployment may lead to increases in abusive behaviour [39, 41, 43]. Corroborating this, previous studies have shown that domestic violence increases in times of economic recession, linked to the financial stress, unemployment and food insecurity and thus the increased potential for conflict in people’s homes [47–49]. Further, economic pressures may also lead to early marriage as an income generating (bride price) or survival strategy (fewer household members). For example, one of the included studies found that families married off their daughters during COVID-19 as a solution to avoid expenses on costly technological devices for online education and additional school fees [45]. Loss of economic resources may also force adolescent girls to engage in transactional sex, which in turn increases the risks of sexually transmitted diseases, unwanted pregnancy, and early marriage [37].

Fourth, PHEs reinforce existing gender norms. Such gender norms include the traditionally assumed roles of women as caregivers both at home and in healthcare settings. Female workers comprise of 70% of the healthcare workforce and provide majority of care and are thus exposed to a high infection risk, while often having little say in the decisions on health service delivery [50]. Furthermore, the load of unpaid work for women and girls at home increases drastically during PHEs, for example through a higher care burden if household members fall sick or if schools are closed [51]. With a higher (health) care burden and reduced economic opportunities during PHEs, women and girls are likely disproportionately affected, relative to men. Some consequences of this are the disempowerment of girls, dependence on parents or partners, which makes it difficult to leave potential abusive relationships, and violence re-victimisation [39, 41–43, 46].

### Risk of bias appraisal

Table 2 presents the quality ratings based on the Joanna Briggs Institute tool that was applied to assess the included cross-sectional studies [34]. The major limitation of most studies was not account for possible confounding factors. Only three studies applied a more robust control for confounding. First, Epstein et al. [41] covered a long observation period by defining drought as precipitation relative to the 29 previous years. Therefore, the exposure variable was less likely correlated with possible socioeconomic and other confounders that may be associated with places that are historically more drought-prone. Second, Cerna-Turoff et al. [40] used a propensity-score matching approach to create matched pairs of individuals who were displaced due to the earthquake and individuals who were not, using pre-earthquake covariates. Third, Zulaika et al. [37] used a causal-comparative design to compare pregnancy and schooling outcomes between girls who experienced school closures (i.e., the “COVID-19 cohort”) and girls who graduated the year prior to the pandemic (i.e., the “pre-COVID-19 cohort”). Apart from these studies, the outcome measures used in the study by Temple and colleagues [38] were downgraded for (1) not having used a previously validated scale, (2) having used only two items to capture dating violence, and for (3) excluding the aspect of emotional violence.

**Table 2:** Critical appraisal for included cross-sectional studies

Study	Sampling	Participants	Exposure	Condition	Confounding	Measures	Analysis
Augusti et al. [42]	x	x	x	x		x	x
Cerna-Turoff et al. [40]	x	x	x	x	x	x	x
Oliveira et al. [46]	x	x	x	x		x	x
Epstein et al. [41]	x	x	x	x	x	x	x
Karp et al. [43]	x	x	x	x		x	x
Kerekes et al. [44]	x	x	x	x		x	x
Sloand et al. [39]	x	x	x	x		x	x
Temple et al. [38]	x	x	x	x			x
Zulaika et al. [37]	x	x	x	x	x	x	x

The coding is based on the Joanna Briggs Institute tool for the appraisal of cross-sectional quantitative studies. The two categories on “Were confounding factors identified?” and “Were strategies to deal with confounding factors stated?” were collapsed into one column and are marked with “x” if reported in the respective study

Included qualitative studies were assessed based on the Critical Appraisal Skills Programme (CASP) appraisal tool (Table 3) [35]. The quality of the studies by Karp et al. [43] and Rahiem [45] was evaluated as high in all categories of the appraisal tool. The only exception for Rahiem [45] was a lack of critical elaboration on the relationship between the researchers and study participants since the manuscript did not present an explicit positionality discussion or reflection. As for Karp et al. [43], the use of in-depth interviews in comparison to focus group discussions for their research question was not justified. Rashid and Michaud [36] study was assessed as less rigorous in comparison to the former studies because the recruitment of participants was not described in sufficient detail, and they only stated that nine girls between 15–19 years-old were selected “randomly” but offered no further explanation on the exact selection procedures or aspects (other than age). In addition, the study’s quality in terms of analysis was assessed as unclear/insufficient due to a lack of information on whether interviews were recorded and transcribed and a lack of information describing how data analysis was carried out, i.e. whether codes and themes were generated based on qualitative content analysis.

**Table 3:** Critical appraisal for included qualitative studies

Study	Recruitment	Data collection method	Relationship between participants and researchers	Ethics	Analysis
Karp et al. [43]	x		x	x	x
Rahiem [45]	x	x		x	x
Rashid and Michaud [36]		x	x	x	

The coding is based on the Critical Appraisal Skills Program (CASP) tool for the appraisal of qualitative studies. We have highlighted five out of ten items of the CASP tool and marked with “x” if reported in the respective study

## Discussion

This review provides a synthesis of existing evidence on how PHEs are associated with three key gender outcomes- early marriage, sexual and reproductive health, and gender-based violence- among adolescent girls. Six studies examined the association between PHEs and GBV, finding that PHEs led to psychological, physical, and sexual abuse (including online sexual abuse) among adolescent girls [39–41, 43, 44, 46]. Two studies found that the SRH needs of girls, including their menstrual hygiene management and access to contraception, faced increased jeopardy during PHEs [36, 37]. One study examined the impacts of the COVID-19 pandemic on child marriage and found that most adolescent girls in Indonesia were not forced to marry, but rather chose to marry to escape from the combined workload of school and home [45]. However, the author highlighted that other reasons like local customs that promote and accept early marriage and economic pressures also underlie child marriages in this context. Lastly, two studies—Temple et al. [38] and Cerna-Turoff et al. [40]—reported a null relationship between PHEs and the examined gender inequality aspects.

We also identified four key mechanisms underlying the above associations. First, PHE-specific response strategies like home confinement, social restrictions, and closure of schools may exacerbate violence risk and deteriorate the mental health of adolescent girls [37, 39, 41]. Second, PHEs may pose a key barrier to accessing healthcare services [36, 39, 41], which can increase the risk of unwanted pregnancies and poor menstrual hygiene management. Third, PHEs reinforce harmful gender norms related to socially ascribed caregiving and income generation roles, which may negatively affect girls’ educational opportunities thus increasing the risks of child marriage and early pregnancy. Fourth, substantial familial economic losses are often the primary impact of PHEs and can cause psychological distress, which may in turn increase abusive behaviours in the household. Insufficient funds may also lead to default on school fee and health insurance payment, which can subsequently impact girls’ education attainment and health outcomes and also increase the risk of early marriages.

Some key gaps still remain when discussing the impact of PHEs. First, our review found generally limited high-quality evidence on the impact of PHEs on the specific age group of adolescent girls, especially with regards to the outcome of child marriages. It is important that future studies disaggregate data on gender and age and record adolescent-specific outcomes to better understand this impact. Second, while some of the included studies have discussed possible underlying pathways leading from PHEs to gender inequality, future studies should aim to explicitly test these mechanisms in a formal mediation analysis or structural equation model. Finally, none of the included studies were explicitly focused on key vulnerable groups of adolescents who may already be facing other forms of adversity caused by their sexuality or physical impediments. Future studies could apply an intersectionality lens to examine the varied effect of PHEs on vulnerable groups like adolescents with disability, LGBTQ + youth, and orphaned adolescents.

We note the following limitations in our review. First, given that more than half of the included studies were conducted in developing countries, which have lower levels of gender inequality already prior to the emergencies, it is difficult to attribute the heightened inequality solely to PHEs. Second, only two studies had samples that were nationally representative, as a result, the other findings are not generalisable to the wider population. Third, most of the information on GBV and SRH were self-reported, which might be prone to under-reporting, especially for such highly stigmatised outcomes. Finally, we examined studies published in English and considered peer reviewed articles only thus excluding possibly valuable evidence from gray literature, such as policy reports and from certain Spanish- or French-focused world regions.

In light of the documented adversity faced by adolescent girls during PHEs, youth-specific response and mitigation programmes are urgently needed in future crises. Some previous studies provide an indication of how effective protection for adolescent girls might look like. The “Empowerment and Livelihood for Adolescents (ELA)” programme targeted at adolescent girls to alleviate the harmful impacts of school closures in Sierra Leone during the Ebola epidemic in 2013–16 might be a useful example [52]. ELA provided safe spaces for girls to socialise with each other and receive vocational training – thereby reducing the time spent with men and the risk of early pregnancies. Another example is the youth empowerment intervention implemented in Bolivia during the COVID-19 pandemic. The intervention trained girls in technical and soft skills, provided sexual education, and job-finding assistance and effectively reduced the prevalence of violence against girls during the COVID-19 lockdown [53]. Another possible success story could be the digitally delivered “Parenting for Lifelong Health” programme, which provided playful parenting resources to build positive parent–child relationships and reduce domestic violence during the pandemic [54, 55]. To counteract the disruptions in healthcare access, the government of Nepal’s “Minimum Initial Service Package (MISP)” could offer a feasible mitigation approach. After the 2015 earthquake destroyed over one third of the maternal and neonatal care facilities [56], MISP organised mobile reproductive health camps, which offered separate adolescent-friendly services. These encouraged GBV-specific referrals and counselling on contraceptive use among adolescent girls [57]. Lastly, previous studies have established that economic strengthening programmes like cash transfers and subsidies are an important strategy to offset the financial damage that PHEs inflict on families [58]. For instance, the “Girl Empower” intervention implemented in post-conflict Liberia led to a significant reduction in child marriage and increased condom use and sexual abstinence among adolescent girls who received cash transfer [59]. Such large-scale income programmes are the need of the hour, especially in low- and middle-income countries, to protect girls’ educational attainment and alleviate the resource constraints faced by them and their families.

## Data Availability

The datasets used and/or analysed during the current study available from the corresponding author on reasonable request.
